# The Emerging Role of Salivary Oxidative Stress Biomarkers as Prognostic Markers of Periodontitis: New Insights for a Personalized Approach in Dentistry

**DOI:** 10.3390/jpm13020166

**Published:** 2023-01-17

**Authors:** Gaia Viglianisi, Gianluca Martino Tartaglia, Simona Santonocito, Mariacristina Amato, Alessandro Polizzi, Marco Mascitti, Gaetano Isola

**Affiliations:** 1 Department of General Surgery and Surgical-Medical Specialties, School of Dentistry, University of Catania, Via S. Sofia 78, 95124 Catania, Italy; gaia.viglianisi@gmail.com (G.V.); simonasantonocito.93@gmail.com (S.S.); amato.mariacristina@hotmail.it (M.A.); alexpoli345@gmail.com (A.P.); 2 Section of Maxillo-Facial Surgery and Dentistry Fondazione IRCCS Cà Granda, Ospedale Maggiore Policlinico, Department of Orthodontics, School of Dentistry, University of Milan, 20122 Milan, Italy; gianluca.tartaglia@unimi.it; 3 Department of Clinical Specialistic and Dental Sciences, Marche Polytechnic University, Via Tronto 10/A, 60126 Ancona, Italy; m.mascitti@staff.univpm.it

**Keywords:** oxidative stress, thioredoxins, superoxide dismutase, inflammation, periodontitis, therapy, precision medicine

## Abstract

Periodontitis is a multifactorial and infective oral disease that leads to the destruction of periodontal tissues and tooth loss. Although the treatment of periodontitis has improved recently, the effective treatment of periodontitis and the periodontitis-affected periodontal tissues is still a challenge. Therefore, exploring new therapeutic strategies for a personalized approach is urgent. For this reason, the aim of this study is to summarize recent advances and the potential of oxidative stress biomarkers in the early diagnosis and personalized therapeutic approaches in periodontitis. Recently, ROS metabolisms (ROMs) have been studied in the physiopathology of periodontitis. Different studies show that ROS plays a crucial role in periodontitis. In this regard, the reactive oxygen metabolites (ROMs) started to be searched for the measures of the oxidizing capacity of the plasma understood as the total content of oxygen free radicals (ROS). The oxidizing capacity of plasma is a significant indicator of the body’s oxidant state as well as homocysteine (Hcy), sulfur amino acid, which has pro-oxidant effects as it favors the production of superoxide anion. More specifically, the thioredoxin (TRX) and peroxiredoxin (PRX) systems control reactive oxygen species (ROS), such as superoxide and hydroxyl species, to transduce redox signals and change the activities of antioxidant enzymes to remove free radicals. Superoxide dismutase (SOD), catalase, and glutathione peroxidase (GPx), among other antioxidant enzymes, change their activity when ROS are produced in order to neutralize free radicals. The TRX system is triggered and transduces redox signals to do this.

## 1. Introduction

Periodontitis is a chronic inflammatory disease that affects the supporting tissues of the dental elements, resulting in loss of periodontal attachment and resorption of alveolar bone, which if left untreated can lead to tooth loss [1]. It is a very common disease, constituting the sixth disease affecting adults worldwide, and is listed as the leading cause of tooth loss in the world. [2]. Periodontitis is referred to as a multifactorial disease in the determination of which several factors contribute, including the presence of periodontopathogenic bacteria, host response, and genetics [3]. In the genetic predisposition patient, the dysbiotic environment allows the development of virulent bacterial communities. These virulent bacterial communities start to stimulate the host immune response [4] recalling the polymorphonucleate cells. Polymorphonucleates take part in the first immune phase and produce reactive oxygen species (ROS) to kill the microorganisms [4]. In periodontitis, it was seen from various studies that the polymorphonucleates are hyperactive. This hyperactivity causes an increase in ROS production and establishes an oxidative stress environment [5,6]. The body possesses various species, called antioxidants, that protect itself from ROS increment. In fact, ROS are produced in a healthy system, but their level is maintained under control thanks to antioxidants [6,7]. The alteration of the balance between antioxidants and ROS causes disease [8,9]. There are secondary factors that take part in the periodontitis process such as diabetes, cardiovascular disease, stress, and smoking tobacco [10,11,12,13]. Diabetes and periodontitis have in common the increase in ROS concentration in the body, while tobacco is an exogenous source of ROS level enhancement [14]. In diabetes patients, the rising of ROS participates in the insulin resistance process [15]. The presence of these factors in periodontopathic patient exacerbate the oxidative stress environment.

The main therapy against periodontitis is scaling and root planning called active therapy, which is performed in all patients. Stop smoking and control the diabetes parameter by taking part in active therapy. After the active therapy, if there are still present 6 mm or more deep pockets and bone defects, it is possible to do periodontal surgeries to solve them [13,16].

This review aims to describe current evidence on the role of ROS and antioxidants in the periodontal disease and underline the possibility to use reactive oxygen metabolisms (ROMs) and antioxidants level in saliva as a new diagnostic tool in periodontitis.

### The Oxidative Stress

Homeostasis is a complex process present in all living systems that controls and regulates system functions in response to the outside environment changes. Involved in this dynamic system are: enzymes, pH, temperature, ions concentration, oxygen concentration and many others [17]. All the cells benefit from homeostasis processes and at the same time [18]. A body is considered healthy when it possesses a balance in homeostasis regulation [17].

**Figure 1:**
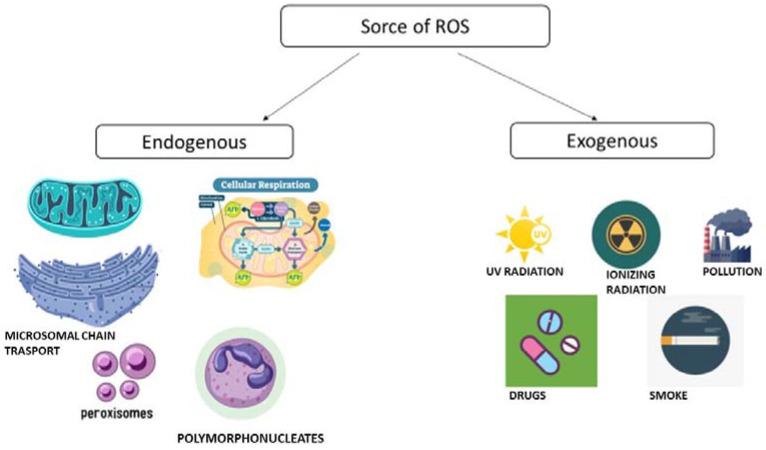
These are the main source of ROS (reactive oxygen species).

In the homeostasis system, the reactive oxygen species (ROS) regulate the balance between health and illness in a living system. ROS are obtained from the redox reactions on O2 molecules [19]. These species originate from many mechanisms such as the oxidation of NADPH, cyclooxygenases, xanthine oxidase, peroxisomes, and microsomal electron transport chain [20]. Moreover, they can be produced from exogenous sources such as pollution, smoke, pesticides, certain drugs such as halothane and paracetamol, UV radiation, and heavy metals [14] (Figure 1).

Superoxide anion (•O2^−^), hydroxyl radical (OH•), single oxygen (1O2), and hydrogen peroxide (H_2_O_2_) belong to the group of reactive oxygen species (ROS) [21]. Superoxide anion and hydroxyl radicals are the main free radicals derived from oxygen. Free radicals are defined as nonstable species [22]. In a healthy system free radicals act as signal elements for different processes. In particular, ROS are able to be second messengers because they can oxidate the thiol group, a group present in the protein. The oxidation of this group changes the protein structure and activates the protein’s function [23,24]. For example, they are released by cells during bacterial infection to call macrophages for killing invader [8,9,25,26] and regulate the genetic materials [27]. On the other hand, when the concentration of the free radicals is too high, they can cause diseases [8,9]. Indeed, in the last few years, the role of ROS in the establishment of various illnesses have been reported in many studies such as cancer, diabetes, neurodegeneration, asthma, reproductive disorders [28,29,30], rheumatoid arthritis [31] and periodontitis [32]. A high concentration of free radicals causes an oxidative stress environment. During this condition, ROS can hurt various cell structures such as DNA, RNA [33], lipids [34], and proteins [35]. In a healthy body, the presence of antioxidant molecules gives a balance.

## 2. Materials and Methods

The articles included in this critical review were identified using the major search engines: Pubmed, Scopus, and Google Scholar. The keywords used in all search engines were as follows: oxidative stress and periodontitis. A total of 1099 articles were found on Pubmed, 19,100 results on Google Scholar, and 55,001, covering the following time frame 1995–2022. Of these articles, 168 were included in the following critic review, after exclusion of duplicate papers and papers that did not fit the criteria for selection of papers. Titles and abstracts for inclusion were reviewed by at least two independent researchers. Full articles were requested for all articles that passed the initial screening. Each full article was evaluated by two researchers for final inclusion/exclusion. In case of disagreement, a third researcher was consulted, and the decision was made by consensus. Initial screening was based on the following criteria: RCTs, cohort studies, case–control studies, and case–series that included at least a sample number of 15, meta-analysis, and systematic review. No studies were excluded based on language of publication.

## 3. Antioxidants in the Oral Cavity

ROS act as biological messengers. Once their function is completed, the antioxidant molecules are able to suppress their high reactivity.

Free radicals, in general, are unstable species that require electrons to achieve stability. To achieve this stability, the antioxidant molecules provide electrons to obtain stable species [21]. These bioactive molecules are present in all the fluids of our body. In saliva, two groups of antioxidants are divided into enzymatic and non-enzymatic. Superoxide dismutase (SOD), catalase (CAT), glutathione peroxidase (GPx), glutathione reductase (GSR), thioredoxin (TRX) [36], and oral peroxidase (OP) belong to the enzymatic group [37]. For instance, superoxide dismutase (SOD) transforms two superoxide anions (•O_2_^−^) in H_2_O_2_ and oxygen [38,39]. Catalase is able to transform H_2_O_2_, produced by SOD, in water and oxygen thanks to the help of manganese or iron, two types of cofactors [38,40]. GPX is capable of transforming H_2_O_2_ in water and lipid peroxides [41]. The oral peroxidase activity is performed by salivary lactoperoxidase (LPO) and myeloperoxidase (MPO). Salivary lactoperoxidase (LPO) converts thiocyanate ions (SCN^−^) and hydrogen peroxide into hypothiocyanite ions (OSCN^−^). These OSCN^−^ ions are capable of killing bacteria by binding their proteins. For this reason, the LPO presence in the saliva promotes oral health, contrasting the development of periodontitis [37]. Myeloperoxidase (MPO) uses similar substrates of LPO for its redox activity. The difference between their redox activity lies in the products obtained (both germicidal ions) [42]. MPO redox products are called hypochlorous acid (HOCl) [43,44]. Another important antioxidant is thioredoxin (TRX). It takes part in the thioredoxin-dependent system with thioredoxin reductase. All these species together perform cell protection with the other antioxidant species [45,46]. TRX performs as an antioxidant because it is able to reduce disulfides, which are present in the oxidized form of peroxiredoxins. Peroxiredoxins are antioxidant enzymes that transform H_2_O_2_ in water [47]. Both TRX and peroxiredoxins participate actively in the removal of ROS (Table 1).

Uric acid, albumin, glutathione, melatonin, bilirubin [36], lactoferrin, vitamin C, vitamin A, and vitamin E take part in the endogenous antioxidant system [48]. The endogenous species perform an important role in redox biology. Uric acid plays more than 70% of the saliva’s antioxidant power thanks to its capability to eliminate free radicals [48]. Uric acid performs a double role according to the environment in which it is present. When there is a hydrophilic contest it plays as an enzyme against ROS. Vice versa in a hydrophobic contest, it behaves as an antioxidant [49]. Melatonin possesses different properties such as antioxidant, antiaging, and anti-inflammatory. In the periodontium, melatonin performs as an antioxidant, and it participates in the immune system mechanisms. Moreover, melatonin takes part in bone homeostasis indirectly regulating osteoblastic activities and stimulating the creation of osteoblastic [50]. In this way, melatonin promotes the new bone apposition and contrasts the resorption. The salivary gland produces it following the circadian rhythm reaching its maximum release between 12:00 and 2:00 a.m. [51]. Another important endogenous antioxidant is glutathione. Glutathione is a potent antioxidant that protects cells from ROS damage. Moreover, glutathione performs as a cofactor for the antioxidant enzymes in removing ROS [52]. The oxidated form of glutathione presents a thiol group that can donate H2. This way, it is possible to transform one molecule of H_2_O_2_ in water [53,54]. This reaction is catalyzed by glutathione peroxidase (GPX) [55].

Antioxidants are also present in the gingival crevicular fluid (GCF). The presence of neutrophils and epithelial cells introduces them. Especially, there are glutathione and SOD [36] that protect the periodontium from the collection of ROS [56].

## 4. The Role of ROS in Periodontal Disease

**Figure 2:**
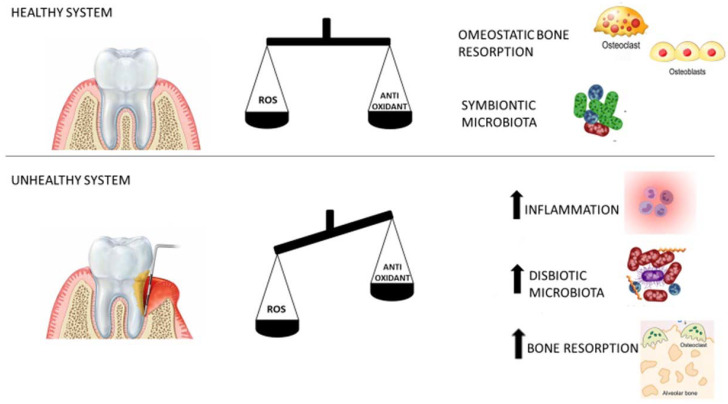
In a healthy system there is a balance between reactive oxygen species (ROS) and antioxidant concentration. This equilibrium allows for maintaining homeostasis in the periodontal tissue structures. If ROS level, for some reason, becomes higher than the antioxidant level, the balance is broken. In that situation, the high level of ROS causes inflammation in the periodontal tissue. At the same time, the microbiota communities change in favor of virulent bacterial communities, this causes the instauration of a dysbiotic microbiota. If this condition is not resolved, it becomes chronic, and the permanence of these mechanisms causes the destruction of the periodontal tissues.

ROS has many roles in the periodontal tissue. During the correct equilibrium of oxidative stress, ROS behave as the cellular messenger, stimulate the production of molecules for the correct function of the cells, and stimulate the immune system to react against pathogens. The presence of pathogens in the periodontal pocket stimulates the release of cytokines, which recall polymorphonucleates to phagocyte the invaders [57]. During the macrophage’s activity, they produce free radicals causing an increase in the concentration of ROS [36]. The ROS level increases in a healthy system allowing the killing of pathogens and promoting the response of the host immune system. In this case, ROS perform a protective role. Unfortunately, when the infection persists, the balance is broken, and the ROS increase becomes a cause of illness [57] (Figure 2).

In different studies, the relationship between periodontitis and ROS was investigated. In this research, the presence of ROMs (reactive oxygen metabolites) in the serum was evaluated. As a result, these studies showed that the ROM level was higher in the periodontopathic patients compared to the healthy ones [58,59,60].

The resorption of the alveolar bone present in periodontitis is based on the alteration of the homeostatic axis. Normally, there is a homeostatic axis that has the role to stimulate the neo-apposition of bone and the resorption of the old one. In periodontitis, the increase in ROS in the periodontal pockets causes the overproduction of cytokines that break the RANKL/osteoprotegerin axis [61,62,63]. When this equilibrium is altered, we have inflammatory bone-related illnesses such as periodontitis, rheumatoid arthritis, osteoarthritis, and osteoporosis [64].

As mentioned before, periodontitis leads to soft tissue distraction. Studies showed that one of the causes of clinical attachment loss is the activation of the metalloproteases (MMPs) caused by ROS oxidation, for instance, hydrogen peroxide [65]. It was seen that when there is an oxidative stress environment, different elements of the periodontium such as collagen, elastin, proteoglycans, and glycosaminoglycans (hyaluronic acid) started to be degraded [66]. Therefore, the oxidated fatty acids trigger adipogenesis and inhibit osteogenesis, causing the periodontium components’ degradation [67]. It was documented that ROS reduces the production of collagen in the cells present in the extracellular matrix [68,69,70,71,72].

The altered concentration of ROS and the material produced by the destruction of periodontal tissue are substrates that stimulate the release of cytokines and the immune system. In this way, it establishes a circuit that feeds the persistence of chronic inflammation. Moreover, in the presence of risk factors, the ROS concentration is higher and exaggerates periodontal disease [73]. Risk factors such as smoking cigarettes, diabetes, and cardiovascular disease have in common the production of ROS and the enhancement of oxidation reactions with periodontitis. Tobacco is an exogenous origin of ROS [14]. In diabetic patients, the resistance to insulin causes a decrease in antioxidants and, as a response to the system, ROS concentration increases [74]. In cardiovascular disease, it was seen an association with periodontitis. The two pathologies have the same risk factors in common; both present an alteration of oxidative stress and inflammation [75]. Furthermore, a new study reported that the type of diet followed can influence the inflammation of the soft tissue around the tooth. This is because diet impacts the oral biofilm in favor of a healthy or an unhealthy environment [76].

## 5. Studies of Possible Biomarker for Periodontitis

Clinical parameters, such as bleeding on probing, clinical attachment level, and others, are important tools for the prognosis of periodontitis. These parameters cannot highlight the beginning of the periodontitis process. For this reason, it is necessary to find predictable parameter variations to diagnose periodontal disease early [77]. Scientists are studying biomarkers that can be used for this goal. The discovery of the relationship between periodontitis and ROS led to the search for reactive oxygen species biomarkers. The shorter half-life and the instability of the ROS do not allow us to use them as biomarkers for periodontitis. Therefore, scientists have focused their attention on terminal products of redox reaction and antioxidant species concentration [78]. These biomarkers are researched in a fluid such as saliva and blood.

### 5.1. ROMs Metabolism Products from ROS Activity

ROS can damage important cell structures such as DNA [33], lipids [34], and proteins [35].

Over the years, different studies were conducted to find the relationship between LPO and periodontitis. It was seen that the LPO concentration in saliva was higher in patients with periodontitis in comparison to healthy patients [79,80,81,82,83,84,85]. From 1995, LPO concentration was suggested to be used as a marker to evaluate the degree of periodontal tissue destruction [86]. Instead of the research on LPO, later studies evaluate the product of LPO activity, malondialdehyde (MDA), a lipid peroxidation biomarker [87]. Dakovic in his study find out how the concentration of MDA in the saliva was related to the grade of inflammation in the periodontium. He concluded by stating that the match of this marker in saliva indicates the presence of inflammatory activity at that moment [88]. Other researchers agree with Dakovic [89,90,91] and the most recent study of 2022 by Veljovic, et al. obtain statistically significant results of the positive relation between periodontitis and MDA level in the blood and saliva [92]. Moreover, in studies by Dacovik [88] and Veljovik [92] it was seen how the scaling and root planning had decreased PD and MDA in the blood and saliva of the periodontal patients. Nonetheless, in the study by Veljovic [92] the smoking impact on the concentration of MDA in the blood of periodontal patients was investigated. Actually, as mentioned before, smoke is one of the exogenous factors that cause the increase in ROS concentration. In periodontal patients tobacco increases the ROS and MDA concentration in saliva and blood, exaggerating the inflammation activity. In another study conducted by Altıngöz et al. in 2021 [93] the relationship between oxidative stress and periodontitis was evaluated in a patient with and without diabetes. The results show that the salivary level of MDA positively correlates with the clinical attachment level (CAL).

An important marker that can be used to evaluate tissue damage in periodontitis is 8-hydroxydeoxyguanosine (8-OHdG). 8-OHdG is a biomarker of oxidative DNA damage. Chen et al. [78] saw an improvement in the concentration of 8-OHdG in the saliva of the patient affected with periodontitis compared to the control group. Altingoz et al. [93] conducted a study to evaluate the OS in periodontopathic patients with and without diabetes. This study showed that 8-OHdG is positively correlated with BOP and CAL as previous studies have seen [94,95]. Moreover, 8-OHdG was the main marker with higher levels in both periodontopathic patients with and without diabetes. The authors suggest the possibility of using 8-OHdG as a new biomarker for the evaluation of periodontal inflammation and for the screening of periodontitis [93]. Sazer et al. [94] and Villa-Correa [95] in their studies, had seen that the level of 8-OHdG was strongly elevated when CAL was upper than 3 mm compared to the sites where CAL was lower than 3 mm. From the literature, CAL is the main parameter for the diagnosis of periodontitis. 8-OHdG could be a new tool to evaluate periodontitis in the future due to its strong correlation with CAL. An interesting study conducted by Almerich-Silla et al. [96] showed the presence of specific bacteria such as Porphyromonas gingivalis, Treponema denticola, and Tannerella forsythia deeply influenced the concentration of 8-OHdG. This strong relationship between this marker and the periodontopathic bacterial underline how the grade of inflammation influenced the level of 8-OHdG in saliva. Önder et al. [84] and Anusuya et al. [97] from their studies have seen that the level of 8-OHdG decreased after non-surgery therapy in periodontopathic patients.

Another biomarker of oxidative stress, derived from the lipid peroxidation, is 4-hydroxy-2-nonenal (4-HNE) [87]. 4-HNE possesses a higher sensitivity (83%) and specificity (81%) for the diagnosis of periodontitis in diabet patients [93]. Onder et al. [84] conducted a study where was seen that the level of 4-HNE not change in the patient with periodontitis compared to the control group. Conversely, Altigoz et al. [93] from their study obtained that 4-HNE was positively correlated with the BOP and CAL. Moreover, the level of 4-HNE was higher in the periodontopathic patient affected by diabetes compared with the periodontopathic patients without diabetes. In a study conducted by Hendek et al. [98] an increase in the level of 4-HNE in the crevicular fluid of smoking periodontopathic patients compared to the non-smoking periodontopathic patients was seen.

A new marker recently studied for the first time in saliva by Altingoz [93] is advanced glycation ends products (AGEs). They derived from the permanent non-enzymatic glycation of proteins and lipids [99] in the presence of an oxidative stress environment. Additionally, they can be introduced into the body from exogenous sources such as UV, smoking tobacco, foods, microwaves, and ultrasounds [100]. The level of AGEs is related to the degree of periodontitis [101,102]. To express their properties AGEs must bind their receptors called RAGE (receptor for advanced glycation end products). When RAGE is activated, AGEs are able to stimulate: the release of cytokines, bone resorption by osteoblastic, the monocytes activities, and collagenase [103]. RAGE is upregulated by the concentration of AGEs. The strong relationship between smoking tobacco and the increase in AGE–RAGE concentration was seen by Katz et al. [104]. They exposed a human fibroblastic culture with 1 μM of nicotine for 72 h. After these hours they obtained more than quadruple the concentration of RAGE in the cultures tested compared to the control group [104]. In Altigoz’s study [93], the AGEs level was significantly higher in patients affected by periodontitis and diabetes in comparison with the control group. Another aspect achieved for the first time in this study was the salivary level of the AGE receptor, RAGEs. It was seen that salivary RAGEs level was lower in the periodontopathic and diabetic patients compared to the control group. Authors concluded underline that RAGEs possess a significant precision in distinguishing between healthy patients and periodontopathic patients [93]. A previous study conducted by Singhal et al. [105] showed that RAGEs serum level was lower in the periodontopathic and diabetic patients compared to the healthy group.

### 5.2. Antioxidants Level in Periodontitis

Two of the antioxidant enzymes that participate in the redox reactions are SOD and GPX. The concentration of SOD and GPX were studied by Chen et al. in 2019 [78] in the saliva and the crevicular fluid of patients with periodontitis and in healthy patients. From the result of this study SOD and GPX do not change their concentration in patients with periodontitis in comparison with the control group. Additionally, Kluknavska et al. [106] in their study, do not see a change in the level of GPx in saliva between patients with and without periodontitis. Other studies [89,107,108,109] obtained opposite results in which SOD and GPX concentrations were altered in the periodontopathic patients. For example, Trivedi et al. [108] have seen a decrease in SOD and GPX activity in the periodontopathic patient. They justify this decrease by explaining the possible damage to the antioxidants during the removal of ROS. In contrast, Trivedi Villa-Corea et al. [110] showed an increase in GPX activity in the group of patients with aggressive and chronic periodontitis compared with the control group. Wei et al. [107] and Yang et al. [111] showed an increase in SOD activity in patients with periodontitis compared with the control groups. Moreover, SOD is positively correlated with the CAL, bleeding on probing (BoP), probing depth (PD), gingival index (GI), and plaque index (PI) [77]. It was seen by Wei et al. [107] and Novakovic et al. [112] that after scaling and root planning the level of SOD diminishes and GPX reaches the concentration present in the control group.

Glutathione, as mentioned before, participates as a scavenger to remove reactive oxygen species. Various studies found a different glutathione concentration in the periodontopathic patient’s saliva. Kluknavska et al. [106], from their study, have seen an increase in glutathione’s level in patients with aggressive and chronic periodontitis. The same results are achieved in previous studies [113,114,115,116]. Moreover, Kluknavska et al. [106] observed that the glutathione level was lower in the gingivitis patients compared to the periodontopathic ones. At the same time, the glutathione level was higher in gingivitis patients than in the control group. From this result, the authors concluded underline how glutathione increases according to the grade of the inflammation present at that moment in the saliva. In contrast with these results, Tsai et al. [81] and Oktay et al. [117] in their studies found a lower level of glutathione in patients with periodontitis compared to healthy patients.

Melatonin is an antioxidant present in saliva that can be used as a biomarker. Its level variation in periodontopathic patients has been studied. Balaji et al. [118] in their study discovered that the level of melanin in the gingival crevicular fluid was lower in periodontopathic smoking patients and periodontopathic patients compared to the healthy group. In addition, the level of melatonin was lowest in the group of smoking patients affected by periodontitis compared to the nonsmoking group. The authors justify these results by the fact that melatonin decreases its concentration carrying out its antioxidant properties due to the high concentration of ROS [118]. Moreover, the reason why the melatonin level was lower in the smoking group is related to the fact that tobacco is an exogenous factor of ROS increasing [118]. According to Balaji, Purrahmani et al. [119] compared the salivary level of melatonin in patients with and without periodontitis before and after the periodontal treatment. At the baseline, the melatonin level was low in patients with periodontitis compared to the healthy group. After the non-surgical treatment, the melatonin level increased in patients affected by periodontitis. Therefore, Purrahmani [119] reported that salivary melatonin level possesses good sensitivity (80%) and specificity (80%) to evaluate the response of the periodontal treatment.

As mentioned previously, uric acid performed more than 70% of the antioxidant activity in saliva [48]. Over the years many studies have been conducted to find a possible relation between the altered uric acid level and periodontitis. The uric acid level in serum and plasma was studied, and it was obtained that the level increased in the periodontopathic patients [120,121,122]. In contrast with this result, Babaei et al. [123] have seen a decrease in the serum level of uric acid in patients affected by periodontitis. at the same time, Narendra et al. [124] did not see any change in the serum level of uric acid between periodontopathic patients and the healthy group. All these studies have evaluated the serum level of uric acid. Other researchers evaluated the salivary acid uric level. Different studies showed that the salivary level of uric acid was lower in patients with periodontitis than in healthy patients [125,126,127,128]. Moreover, Priya et al. [125] evaluated the salivary uric acid level variation after the non-surgical periodontal treatment. This study showed that salivary uric acid increased in periodontopathic patients after the non-surgical treatment. This result was also gained by Sayar et al. [129] and Baz et al. [130]. The non-surgical treatment allows for a decrease in the concentration of bacterial and oxidative stress products. The removal of bacterial and oxidative products permits the increase in uric acid to the level of healthy patients [131]. Contrarily, Mathur et al. [132] did not see any increase in the salivary uric acid level in patients affected by periodontitis after the periodontal non-surgical treatment. Priya et al. [125] concluded that uric acid level and arginase could be biomarkers to evaluate the early degree of inflammation and the healing obtained from the periodontal treatment. This is because a positive correlation between arginase and uric acid with CAL and PPD was seen. Unfortunately, this positive correlation with periodontal parameters is maintained until the 90th day after the treatment [125].

### 5.3. Total Status and Capacity of Antioxidants

Instead of investigating the concentration in saliva of only one marker, in 2005, Erel et al. [133] developed an automatic colorimetric system for the evaluation of the total oxidant status (TOS). During the years, this system was used in many studies to investigate the oxidant status in periodontitis patients. There are different opinions in the literature about the use of TOS as biomarkers in periodontitis. In many researchers, TOS level resulted to be higher in the periodontopathic patients compared to the control group [107,134,135,136]. Despite the result obtained, Toczewska et al. [134] claimed that TOS could not be used for the diagnosis of the stage of periodontitis in patients. Moreover, Zalewska et al. [137] have seen that the level of TOS was higher in the stimulated saliva compared to the non-stimulated saliva and CGF. They justify this result by explaining that parotids are the principal source of ROS in the mouth. According to the results of Wei et al. [107], Baltacıo˘glu et al. [135], and Toczewska et al. [134] obtained from their study, the level of TOS in patients with periodontitis decreased after the non-surgical treatment. This underlines how the salivary level of TOS may increase during periodontitis due to the increase in ROS concentration. On the contrary, in their study, Zhang et al. [138] did not notice a different level of TOS between healthy patients and periodontopathic patients. However, this result may have been altered due to the high presence of smoker patients as tested and controller cases [77]. In different clinical trials, it was seen a decrease in the total antioxidant status in patients with periodontitis at the beginning of the studies. After the non-surgical treatment, the level of total antioxidants improved in the same patients [139,140,141,142].

Another marker studied is TAOC (total antioxidant capacity), a parameter used to indirectly evaluate the activity of antioxidants against oxidative stress in periodontitis. Baser [143], Miricescu [109], and Nguyen [144] have seen a low TAOC level in patients affected by periodontitis compared to the control group. The decrease in the total level of antioxidants in saliva may be an effect of antioxidant activity against increasing ROS [145]. On the other hand, there is a study where the level of TAOC increased in CFG and saliva in patients with periodontitis compared to the control group [146]. These contrasting studies are related to the fact that at the beginning of the disease, there is an increase in the TAOC level in saliva. However, when the mechanism of the disease becomes chronic and the ROS level increases, the TAOC level becomes lower. Investigating the level of TAOC may be better than evaluating the level of each antioxidant. Moreover, the level of TAOC gives us a reflection of the oxidant status in the environment of the mouth of the patient at that moment [145].

A further parameter called total antioxidant capacity (TAC) has been studied as a new biomarker in periodontitis. Several studies observed a decrease in the TAC level in patients affected by periodontitis compared to the control group [134,138,143,147,148]. Zhang et al. [138] and Senouci et al. [148] reported a negative correlation between TAC and the grade of periodontitis. Moreover, they identified a positive correlation between TAC and CAL [138,148]. These results justify the outcomes obtained by Novakovic et al. [149] and Behfarnia et al. [150], which have increased TAC levels after non-surgical treatment in patients affected by periodontitis. On the other hand, another study did not report a difference between the TAC level in stimulated and non-stimulated saliva [134]. The TAC biomarker was used in a study in which was evaluated the oxidative stress in children and adolescents affected by caries. The authors divided the patients into two groups, the first group with children aged from 3 to 18 and the second group with adolescents aged from 13 to 18.

From this study, it was discovered that in the first group, patients with papillary bleeding probing (PBI) presented an increase in the TAC level. The authors justify these results by saying that the increase in antioxidants was made to contrast the ROS increase. At the same time, in the second group, the TAC level decreased in presence of an oxidative stress environment caused by severe dental caries. In these cases, the authors stated that the TAC decrease was caused due to the utilization of antioxidant defense against the ROS increase. It is important to say that these conflicting results can be related to the immaturity of the immune system in these patients [151]. Despite some good results, TAC and TOS are not reliable parameters to differentiate the periodontitis grade and diagnose periodontal disease. The increase in these parameters can be caused by various inflammation conditions [134].

### 5.4. Biomarkers Level in Patients with Periodontitis and Systemic Disease

Over the years, experts have seen an association between periodontitis and different systemic diseases such as diabetes, cardiovascular disease, pregnancy complications [11,152,153], metabolic disease, obesity, rheumatoid arthritis, and respiratory disease, and others are still under study [154]. This correlation is based on the fact that the inflammation that characterized periodontitis, influenced the systemic immune responses of the other pathologies [11,152,153].

Various studies started to search the biomarkers of oxidative stress levels in the fluid of patients affected by periodontitis and other diseases. In diabetic patients, it was seen that the level of MDA, a product of lipid peroxidation, increased in patients affected by type 2 diabetes. Moreover, it was seen that the level of antioxidants decreases in diabetic patients compared to the control group [155]. A recent study analyzed the level of MDA in different groups affected by periodontitis, periodontitis and diabetes, periodontitis and smoking cigarettes and periodontitis, diabetes and smoking cigarettes. At the end of the study, the results evidenced how the presence of more than one condition exaggerates the oxidative stress environment. In fact, the level of MDA increases in all the groups but the group with all three conditions possess the higher level of MDA [156]. The level of MDA was higher compared to healthy patients and also in patients affected by periodontitis and heart disease [157]. The inflammation present in bowel inflammation diseases such as Crohn’s disease impacts the salivary glands and causes the production of oxidative stress biomarkers in saliva [158]. Consequently, researchers started to study the saliva of patients affected by these groups of diseases to find oxidative stress biomarkers. In a study conducted by Jansakova et al., it was seen that the antioxidant level decreased in patients affected by Crohn’s disease compared to the control group. Moreover, in this study, an increase in AGEs levels in patients affected by Crohn’s disease was seen [159]. The increase in AGE levels is also common in patients affected by periodontitis and diabetes [160].

Other biomarkers that change their level in serum and saliva in patients affected by periodontitis and other disease are vitamins D and C. Normally, these two vitamins play as antioxidants. In particular, vitamin D is able to stimulate SOD and glutathione peroxidase activities in the fat tissue [161] Moreover, it can minimize the production of ROS during cellular respiration in the fat cells [162]. In a recent study conducted by Li et al. [163] the serum level of vitamins in patients affected by periodontitis and metabolically unhealthy overweight and obesity was evaluated. From this study, it was seen that in these patients the plasma level of vitamin C and D decreased. This result underlines how these two antioxidants play an important role in the mechanism of defense against the increase in ROS in the human body [163]. The variation in the level of vitamin C causes the shift of the oral biofilm and the establishment of an oxidant stress environment [164]. Another study conducted by Isola et al. [165] evaluated the saliva and serum levels of vitamin C and antioxidants in patients affected by periodontitis and ischemic heart disease. From the results of this study, the levels of antioxidants and vitamin C were lower in patients affected with both diseases compared to the patients affected only by periodontitis or in healthy patients [165]. According to the results obtained by Isola et al. and Noshin et al. [157] a decrease in the antioxidant level in cardiovascular patients was seen. These studies underline how the coexisting of periodontitis and other pathologies cause the exaggeration of the oxidative stress level with the result of a decrease in antioxidant concentration in saliva and plasma. Further studies are necessary for a better understanding of the variation of the oxidative stress biomarkers level in periodontopathic patients with other conditions.

## 6. Future Perspectives

Nowadays, clinical attachment loss (CAL) is used as the main tool for the diagnosis of periodontitis [165]. However, CAL can only be encountered when the disease is already established. For this reason, over the years scientists have focused their attention on the research of new tools for the early diagnosis of periodontitis before the initial loss of teeth tissue. After the relationship between oxidative stress and periodontitis has been discovered, researchers have begun searching for biomarkers related to oxidative stress products present in saliva [78]. Saliva is rich in molecules that reflect the system condition. Moreover, saliva collection is an easy and non-invasive process [166]. Due to these characteristics, the detection of salivary biomarkers could be an important tool for the early detection of periodontitis that will benefit the population [167,168] and will play an important role in future studies.

## 7. Conclusions

In conclusion, reactive oxygen species (ROS) play a first-order role in the development and progression of periodontitis. The presence of high levels of ROS in periodontitis may compromise the balance between ROS and antioxidants; in this case, the activities of antioxidants can no longer cope with the increase in ROS. The detection of metabolites created in the context of oxidative stress in saliva appears to be possible biomarkers for early diagnosis of periodontitis. However, further studies are still needed in order to better understand the sensitivity and specificity of these salivary biomarkers and to provide more opportunities and tools to further understand and support the early diagnosis of periodontitis.

## Figures and Tables

**Table 1:** List of the antioxidants enzymes and their reaction to remove reactive oxygen metabolites (ROMs).

Antioxidant Enzymes	Mechanism	Ref
Superoxide dismutase (SOD)	Transforms 2O_2_^−^ in H_2_O_2_ and O_2_	[36,37]
Catalase (CAT)	Transforms H_2_O_2_ in H_2_O and O_2_, in presence of manganese or iron	[36,38]
Glutathione peroxidase (GPX)	Transforms H_2_O_2_ and 2GSH in 2H_2_O and oxidated form of GSH	[39]
Salivary lactoperoxidase (LPO)	Convert H_2_O_2_ and SCN^−^ into OSCN^-^ and H_2_O	[35]
Myeloperoxidase (MPO)	H_2_O_2_ e chloride ions (Cl^−^) into hypochlorite acid (HOCl)	[41,42]
Thioredoxin and peroxiredoxins	Participate in a redox circle for the transformation of H_2_O_2_ into H_2_O and O_2_	[45]

## Data Availability

Data are available from the corresponding author upon reasonable request.

## Abbreviations

Reactive oxygen species	ROS
Reactive oxygen metabolisms	ROMs
Catalase	CAT
Superoxide dismutase	SOD
Glutathione peroxidase	GPX
Glutathione reductase	GSR
Thioredoxin	TRX
Oral peroxidase	OP
Salivary lactoperoxidase	LPO
Clinical attachment level	CAL
Bleeding on probing	BoP
Probing depth	PD
Gingival index	GI
Plaque index	PI
Periodontal probing depth	PPD
Myeloperoxidase	MPO
Malondialdehyde	MDA
Hydroxydeoxyguanosine	8-OHdG
4-hydroxy-2-nonenal	4 HNE
Advanced glycation ends products	AGEs
AGE receptor	RAGE
Total oxidant status	TOS
Crevicular gingival fluid	CGF
Total antioxidant capacity	TAOC
Total antioxidant capacity	TAC
