# Correction: Viglianisi et al. The Emerging Role of Salivary Oxidative Stress Biomarkers as Prognostic Markers of Periodontitis: New Insights for a Personalized Approach in Dentistry. *J. Pers. Med.* 2023, *13*, 166

**DOI:** 10.3390/jpm15060243

**Published:** 2025-06-10

**Authors:** Gaia Viglianisi, Gianluca Martino Tartaglia, Simona Santonocito, Mariacristina Amato, Alessandro Polizzi, Marco Mascitti, Gaetano Isola

**Affiliations:** 1 Department of General Surgery and Surgical-Medical Specialties, School of Dentistry, University of Catania, Via S. Sofia 78, 95124 Catania, Italy; 2 Section of Maxillo-Facial Surgery and Dentistry Fondazione IRCCS Cà Granda, Ospedale Maggiore Policlinico, Department of Orthodontics, School of Dentistry, University of Milan, 20122 Milan, Italy; 3 Department of Clinical Specialistic and Dental Sciences, Marche Polytechnic University, Via Tronto 10/A, 60126 Ancona, Italy

Following discussions between the Editorial Board and the authors, the original published references no. 29, 68, 69, 71, and 72 in the original publication [1] have been removed and replaced by the following:

29. Chávez, M.D.; Tse, H.M. Targeting Mitochondrial-Derived Reactive Oxygen Species in T Cell-Mediated Autoimmune Diseases. *Front. Immunol.* **2021**, *12*, 703972. https://doi.org/10.3389/fimmu.2021.703972.
68. Ewald, C.Y. Redox Signaling of NADPH Oxidases Regulates Oxidative Stress Responses, Immunity and Aging. *Antioxidants* **2018**, *7*, 130. https://doi.org/10.3390/antiox7100130.
69. Huang, H.; Du, W.; Brekken, R.A. Extracellular Matrix Induction of Intracellular Reactive Oxygen Species. *Antioxid. Redox Signal.* **2017**, *27*, 774–784. https://doi.org/10.1089/ars.2017.7305.
71. Ahamed, J.; Laurence, J. Role of Platelet-Derived Transforming Growth Factor-β1 and Reactive Oxygen Species in Radiation-Induced Organ Fibrosis. *Antioxid. Redox Signal.* **2017**, *27*, 977–988.
72. Roberts, D.D. Extracellular Matrix and Redox Signaling in Cellular Responses to Stress. *Antioxid. Redox Signal.* **2017**, *27*, 771–773. https://doi.org/10.1089/ars.2017.7308.

Corresponding updates have also been made to the References section and to the citations throughout the manuscript. The authors state that the scientific conclusions are unaffected. This correction was approved by the Academic Editor. The original publication has also been updated.
